# Repetitive Transcranial Magnetic Stimulation in Stroke Rehabilitation: A Bibliometric Review

**DOI:** 10.7759/cureus.79509

**Published:** 2025-02-23

**Authors:** Ayesha Juhi, Rintu K Gayen, Shreya Sharma, Pritam K Choudhary, Himel Mondal

**Affiliations:** 1 Physiology, All India Institute of Medical Sciences, Deoghar, IND; 2 Electronics and Communication Engineering, Institute of Engineering and Management, Kolkata, IND; 3 Neuromodulation Laboratory, Physiology, All India Institute of Medical Sciences, Deoghar, IND

**Keywords:** bibliometrics, brain, cognition, cortical excitability, magnetic phenomena, neuronal plasticity, review literature as topic, sensation, stroke rehabilitation, transcranial magnetic stimulation

## Abstract

Stroke is a major cause of disability globally, with rehabilitation playing a crucial role in restoring lost functions. Despite advancements, many stroke survivors face persistent deficits, prompting the need for innovative approaches such as repetitive transcranial magnetic stimulation (rTMS). This non-invasive technique promotes neural plasticity and recovery by modulating cortical excitability, garnering significant research interest. This bibliometric analysis of rTMS research in stroke rehabilitation was conducted to find publication trends and influential studies. Data were collected from the Web of Science (WOS) with search strings as follows: TI = ((repetitive transcranial magnetic stimulation) OR rTMS) AND TI = ((stroke) OR stroke rehabilitation). The studies till the 31st of December 2024 were included. No language or other filters were applied. A total of 556 studies were identified. While analyzing the data, there may be a higher or lower count of the total number of studies due to the overlap of categories. For example, a study may have authors from different countries, making the total number of publications according to countries higher than 556. There was a growing interest in rTMS in the context of stroke rehabilitation, with a substantial increase in publications in 2022, 2023, and 2024. Among the studies, the majority of the studies were research articles (62.42%), followed by meeting abstracts (18.41%). The studies (n = 983) were in the fields of clinical neurology (27.47%) and neuroscience (27.37%), followed by rehabilitation (8.55%). When studies (n = 645) were categorized according to countries, The People's Republic of China had the majority of the studies (29.92%), followed by South Korea (11.01%), the USA (10.85%), and Japan (9.61%). Elsevier (15.83%) leads in publishing the articles, followed by Frontiers Media (13.49%). The top citation was for the article titled "Repetitive Transcranial Magnetic Stimulation of Contralesional Primary Motor Cortex Improves Hand Function After Stroke" with 521 citations and was published in the journal Stroke. These findings provide valuable insights into research trends, influential studies, and global collaboration, emphasizing the potential of rTMS in advancing stroke recovery. More studies are needed from diverse geographical regions with possible international collaboration.

## Introduction and background

Stroke is a leading cause of disability worldwide, affecting millions of individuals annually and imposing a significant socioeconomic burden on healthcare systems. Currently, 110 million people worldwide suffer from stroke, with over 60% of those individuals being under 70 years of age [1]. Rehabilitation is a cornerstone of stroke recovery, aiming to restore motor, cognitive, and sensory functions to improve the patient's quality of life [2]. Despite advancements in conventional rehabilitation methods, many stroke survivors experience persistent deficits, highlighting the need for innovative approaches [3].

Repetitive transcranial magnetic stimulation (rTMS), a non-invasive neuromodulation technique, has emerged as a promising adjunct in stroke rehabilitation [4]. By delivering magnetic pulses to targeted brain regions, rTMS can modulate cortical excitability and promote neural plasticity, facilitating recovery of motor and cognitive functions [5]. Over the past two decades, a growing body of research has investigated the therapeutic potential of rTMS in post-stroke rehabilitation, examining its efficacy, optimal protocols, and underlying mechanisms. The growing interest in rTMS for stroke rehabilitation has resulted in a wealth of published literature, including clinical trials, narrative reviews, systematic reviews, and meta-analyses, all exploring its efficacy, mechanisms, and optimal protocols [6].

A bibliometric analysis serves as a powerful tool to evaluate the scientific landscape, identify research trends, and highlight influential studies, authors, and institutions [7]. By systematically analyzing the publication patterns and citation dynamics, such a review can provide a broader perspective on the evolution of rTMS research in stroke rehabilitation. The major limitation of bibliometric analysis is that it focuses on quantitative aspects such as publication counts, citations, and impact factors, which may not fully capture research quality, clinical relevance, or actual scientific contributions. Additionally, it is influenced by database coverage. For example, if a journal is not indexed in Medline, PubMed Central, or Bookshelf and reviewers use only PubMed search in their analysis, they may miss the articles published in that journal.

A previous study by Li et al. reported trends up to 2023 [6], using a search across all fields and including transcranial magnetic stimulation (searched as “TMS”). In the present study, we focused specifically on rTMS in stroke rehabilitation up to 2024, limiting the search to titles. This approach was chosen to ensure that the studies or articles addressing the applicability of rTMS in stroke rehabilitation included these key terms in their titles. If all fields are chosen, then any article having the terms even in the discussion of any research paper may appear in the search result, which is not in the scope of this bibliometric analysis.

## Review

Methods

Database

The Web of Science (WOS) database was chosen for its comprehensive coverage of high-quality, peer-reviewed research publications and its advanced bibliometric tools, which facilitate detailed trend analysis [8]. Additionally, WOS provides reliable citation metrics and robust filtering options. Its credibility and wide acceptance in academic and research communities further make it a preferred choice for bibliometric analyses [9].

Search Strategy 

The following search query was employed: TI = ((repetitive transcranial magnetic stimulation) OR rTMS) AND TI = ((stroke) OR stroke rehabilitation). The search was limited to the title field to ensure that only highly relevant publications were retrieved. The studies until the 31st of December 2024 were included, allowing the inclusion of all available studies up to the search date.

Data Check

To ensure the relevance and quality of the extracted data, two independent authors screened the retrieved articles. Titles and abstracts were carefully reviewed to confirm alignment with the research scope, specifically focusing on the application of rTMS in stroke rehabilitation. Both authors found that all 556 studies were included in the final analysis.

Data Extraction

From the final set of eligible articles, data were extracted on key bibliometric indicators, including publication year, type of publication, WOS category of the publication, country, affiliations, publishers, and citation counts.

Data Analysis

Descriptive statistics were used to summarize publication and citation trends over time. Mean, standard deviation, median, and quartile were used to present the data where necessary. Co-authorship networks and keyword co-occurrence analysis were performed on VOSviewer to highlight collaborative relationships and key research themes.

Results

**Figure 1:**
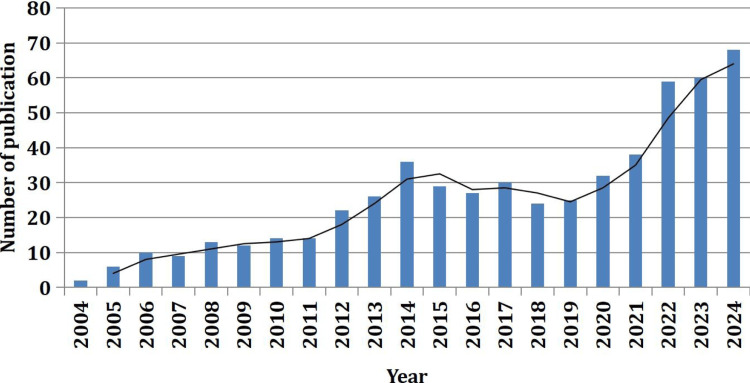
Year-wise number of publication on repetitive transcranial magnetic stimulation in stroke rehabilitation sourced from Web of Science

Among 556, there was a substantial increase in publications in 2022 (59 or 10.61%), 2023 (60 or 10.79%), and 2024 (68 or 12.23%). Publications from 2004 to 2024 are shown in Figure 1.

The growth was relatively gradual from 2004 to 2011, with publication numbers remaining below 20 per year, followed by a more noticeable increase in 2014. The publication increased to its highest in 2024.

**Figure 2:**
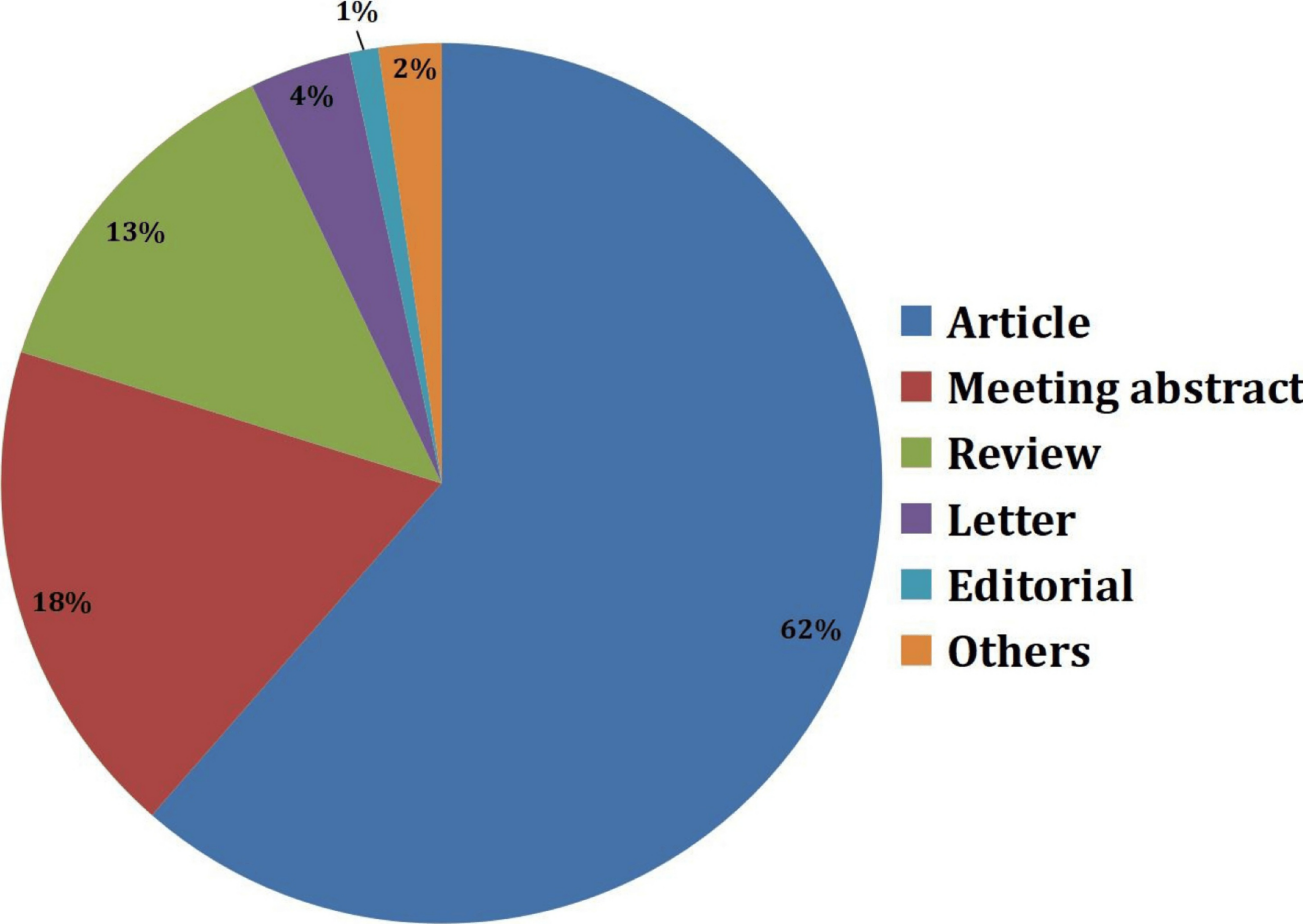
Category of articles sourced from Web of Science

According to the type of article (565 articles categorized into 10), research articles dominate (347 or 61.45%) of all publications. Meeting abstracts represent the second largest category, followed by reviews. The remaining publication types make up a relatively small portion, as shown in Figure 2.

**Figure 3:**
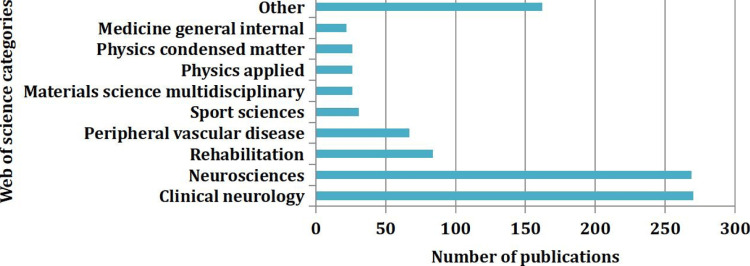
Number of publications according to Web of Science categories

Corrections, early access, retractions, chapters, or proceedings are categorized into others. Figure 3 illustrates the distribution of publications (983 articles categorized into 53) across different scientific categories (n = 53), with clinical neurology and neurosciences being the dominant category, having 270 (27.47%) and 269 (27.37%) publications, respectively.

There is a substantial gap between these leading categories and the rest, with rehabilitation and peripheral vascular disease forming a middle tier with 84 (8.54%) and 67 (6.82%) publications, respectively. A category whose contribution is below 2%, such as medicine research experimental, psychology, psychiatry, and geriatrics gerontology, is combined with others.

**Figure 4:**
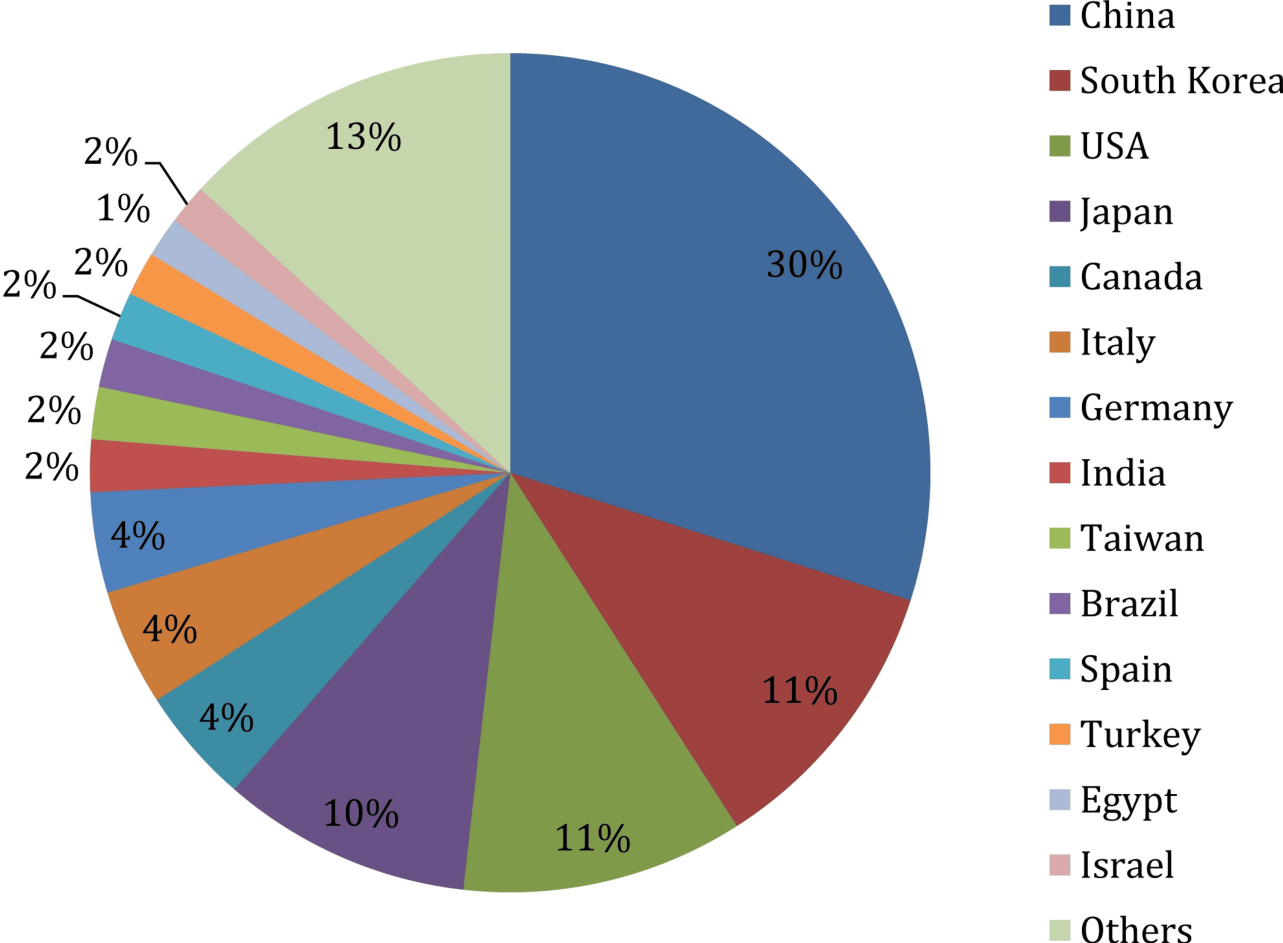
Country-wise distribution of published articles

The country-wise distribution (645 articles grouped into 46 countries) of studies reveals that China leads with the highest contribution (193 or 29.92%), followed by South Korea (71 or 11.01%) and the USA (70 or 10.85%). The distribution is shown in Figure 4.

Any countries with less than 10 publications are added together in others. A country published a median of 3.5 (Q1-Q3: 1-10.75) articles.

**Figure 5:**
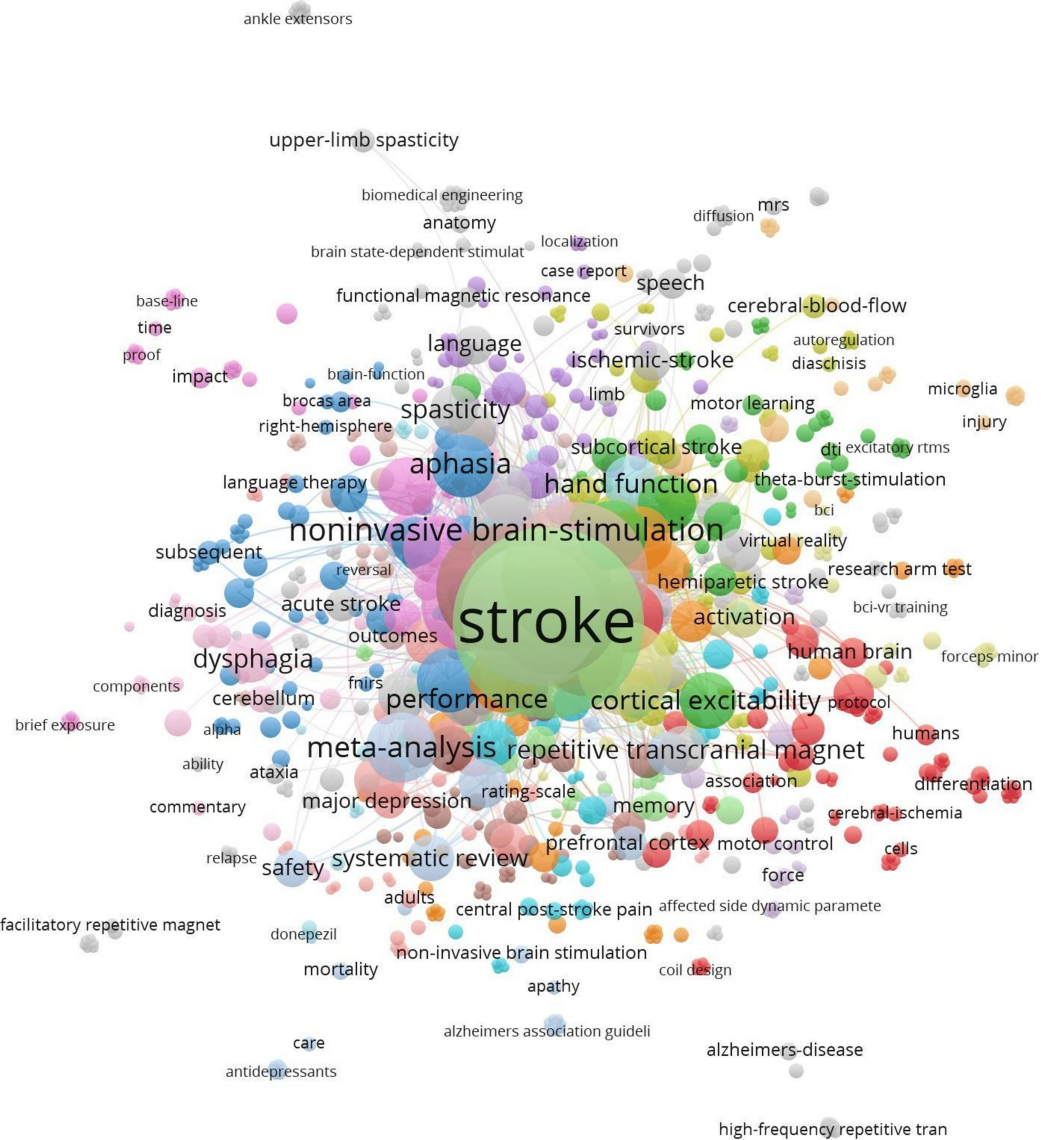
Keyword co-occurrence network visualization in VOSviewer

The keyword co-occurrence analysis highlights stroke as the most frequently occurring term with 302 mentions and the highest total link strength (3,132), emphasizing its central role in the research domain. The network is shown in Figure 5.

Other prominent keywords include transcranial magnetic stimulation (178 occurrences, link strength 1,866) and repetitive transcranial magnetic stimulation (161 occurrences, link strength 1,631), reflecting their importance in stroke rehabilitation research. Related terms such as rehabilitation, recovery, and cortex also demonstrate strong link strengths, indicating their interconnectedness with the primary topics. Emerging terms such as plasticity, low-frequency rTMS, and theta-burst stimulation suggest evolving areas of focus.

The top cited article on the topic was published by Takeuchi et al. in the journal Stroke and was titled “Repetitive transcranial magnetic stimulation of contralesional primary motor cortex improves hand function after stroke.” The top 20 publications are listed in Table 1. 

**Table 1:** Top 20 papers according to citation count in all databases WOS: Web of Science, rTMS: repetitive transcranial magnetic stimulation

Author	Title	Journal	Type	References	Citation (WOS)	Citation (all database)
Takeuchi et al. [10]	Repetitive Transcranial Magnetic Stimulation of the Contralesional Primary Motor Cortex Improves Hand Function After a Stroke	Stroke	Article	20	446	521
Mansur et al. [11]	A Sham Stimulation-Controlled Trial of rTMS of the Unaffected Hemisphere in Stroke Patients	Neurology	Article	10	429	495
Fregni et al. [12]	A Sham-Controlled Trial of a 5-Day Course of Repetitive Transcranial Magnetic Stimulation of the Unaffected Hemisphere in Stroke Patients	Stroke	Article	15	388	430
Khedr et al. [13]	Therapeutic Trial of Repetitive Transcranial Magnetic Stimulation After Acute Ischemic Stroke	Neurology	Article	10	356	428
Kim et al. [14]	Repetitive Transcranial Magnetic Stimulation-Induced Corticomotor Excitability and Associated Motor Skill Acquisition in Chronic Stroke	Stroke	Article	26	358	407
Grefkes et al. [15]	Modulating cortical connectivity in stroke patients by rTMS assessed with fMRI and dynamic causal modeling	Neuroimage	Article	63	262	294
Hsu et al. [16]	Effects of Repetitive Transcranial Magnetic Stimulation on Motor Functions in Patients With Stroke A Meta-Analysis	Stroke	Article	54	242	284
Nowak et al. [17]	Effects of Low-Frequency Repetitive Transcranial Magnetic Stimulation of the Contralesional Primary Motor Cortex on Movement Kinematics and Neural Activity in Subcortical Stroke	Archives of Neurology	Article	30	233	265
Ameli et al. [18]	Differential Effects of High-Frequency Repetitive Transcranial Magnetic Stimulation Over Ipsilesional Primary Motor Cortex in Cortical and Subcortical Middle Cerebral Artery Stroke	Annals of Neurology	Article	48	215	256
Kirton et al. [19]	Contralesional Repetitive Transcranial Magnetic Stimulation for Chronic Hemiparesis in Subcortical Paediatric Stroke: A Randomised Trial	Lancet Neurology	Article	38	169	202
Khedr et al. [20]	Long-Term Effect of Repetitive Transcranial Magnetic Stimulation on Motor Function Recovery After Acute Ischemic Stroke	Acta Neurologica Scandinavica	Article	19	157	198
Avenanti et al. [21]	Low-Frequency rTMS Promotes Use-Dependent Motor Plasticity in Chronic Stroke: A Randomized Trial	Neurology	Article	37	166	186
Khedr et al. [22]	Treatment of Post-stroke Dysphagia with Repetitive Transcranial Magnetic Stimulation	Acta Neurologica Scandinavica	Article	21	145	184
Khedr et al. [23]	Role of 1 and 3 Hz Repetitive Transcranial Magnetic Stimulation on Motor Function Recovery After Acute Ischaemic Stroke	European Journal of Neurology	Article	28	149	182
Takeuchi et al. [24]	Inhibition of the Unaffected Motor Cortex by 1 Hz Repetitive Transcranial Magnetic Stimulation Enhances Motor Performance and Training Effect of the Paretic Hand in Patients with Chronic Stroke	Journal of Rehabilitation Medicine	Article	27	157	179
Weiduschat et al. [25]	Effects of Repetitive Transcranial Magnetic Stimulation in Aphasic Stroke: A Randomized Controlled Pilot Study	Stroke	Article	46	140	165
Hao et al. [26]	Repetitive Transcranial Magnetic Stimulation for Improving Function After Stroke	Sao Paulo Medical Journal	Editorial	1	146	163
Chang et al. [27]	Long-Term Effects of Rtms on Motor Recovery in Patients After Subacute Stroke	Journal of Rehabilitation Medicine	Article	35	134	159
Lefaucheur [28]	Stroke Recovery Can Be Enhanced by Using Repetitive Transcranial Magnetic Stimulation (rTMS)	Clinical Neurophysiology	Review	107	118	146
Dionisio et al. [29]	The Use of Repetitive Transcranial Magnetic Stimulation for Stroke Rehabilitation: A Systematic Review	Journal of Stroke & Cerebrovascular Diseases	Review	91	119	145

The papers had a median citation of 6 (Q1-Q3: 0-28), while 155 had 0 citations, 37 had one citation, and 27 had three citations. A total of 40 papers had more than 100 citations.

**Figure 6:**
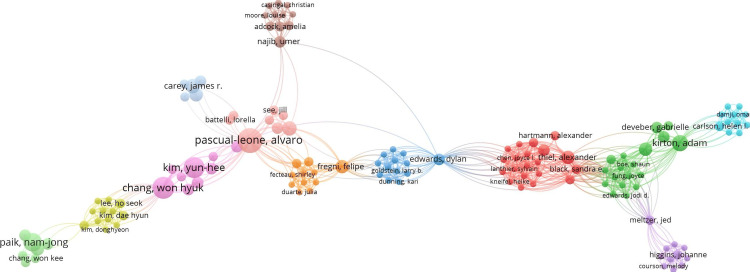
Co-authorship network visualization in VOSviewer

There were a total of 2,450 authors, and their network is visualized in Figure 6.

The author's network analysis highlights Masahiro Abo and Wataru Kakuda as central figures, with the highest total link strengths (174 and 122, respectively), reflecting their strong collaborative roles. Abo leads with 37 documents and 833 citations, followed by Kakuda with 25 documents and 741 citations.

The top institution was Jikei University situated in Minato, Japan. The institutions with high output are shown in Table 2. 

**Table 2:** Top institutions contributing articles

Institutions	Number	Percentage
Jikei University	41	4.36
Harvard University	21	2.23
Beth Israel Deaconess Medical Center	15	1.59
Capital Medical University	15	1.59
Harvard Medical School	15	1.59
Sungkyunkwan University Skku	15	1.59
Samsung Medical Center	14	1.49
Fudan University	11	1.17
Nanjing Medical University	11	1.17
Seoul National University Snu	11	1.17
All India Institute of Medical Sciences, New Delhi	10	1.06
Ben Gurion University	10	1.06
Egyptian Knowledge Bank Ekb	10	1.06
Kangwon National University	10	1.06
Shimizu Hosp	10	1.06
Others	722	76.73

A total of 200 institutions contributed to the articles (n = 941), with a median of 4 (Q1-Q3: 3-5) articles. Any institutions with less than 10 publications are categorized into other categories.

**Figure 7:**
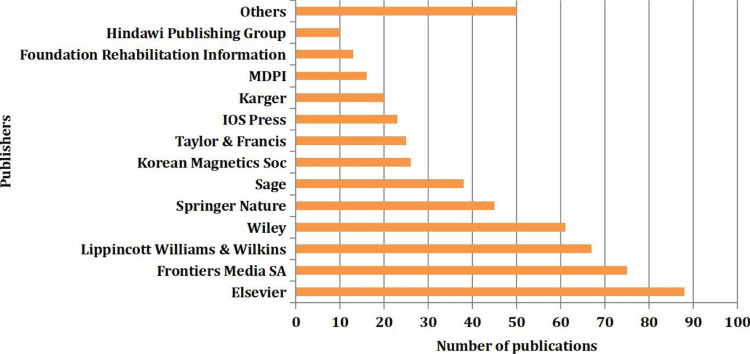
Distribution of publications across various publishers

Figure 7 presents the distribution of publications across various publishers, with Elsevier clearly dominating the field with 88 (15.83%) publications.

Following Elsevier, there is a second tier of major publishers, including Frontiers Media SA (75 or 13.49%) and Lippincott Williams & Wilkins (67 or 12.05%). Any publishers whose contribution was below 10 publications were combined into the “Others” category. A total of 40 publishers published a median of 2.5 (Q1-Q3: 1-17) articles.

A total of 155 journals published the articles with a median of 1 (Q1-Q3: 1-3.5) per publisher. The major contributing journals are shown in Table 3.

**Table 3:** Top journals contributing articles

Journals	Number of publications	Percentage
Frontiers in Neurology	42	8.14
Stroke	28	5.43
European Journal of Neurology	26	5.04
Journal of Magnetics	26	5.04
International Journal of Stroke	20	3.88
Brain Stimulation	17	3.29
Neurology	15	2.91
Cerebrovascular Diseases	14	2.71
Restorative Neurology and Neuroscience	14	2.71
Journal of Rehabilitation Medicine	13	2.52
Clinical Neurophysiology	12	2.33
Neurological Sciences	11	2.13
Neurorehabilitation and Neural Repair	11	2.13
Frontiers in Human Neuroscience	10	1.94
Others	299	57.95

The distribution of publications across journals shows Frontiers in Neurology as the leading publication (42 or 8.14%). Any journals whose contribution was below 10 articles are combined in the “Others” category. A total of 85 journals published only one article.

Discussion

The upward trajectory of publications on rTMS in stroke rehabilitation from 2004 to 2024 reflects the growing interest and recognition of its potential in neurorehabilitation. The modest growth in the earlier years might be attributed to initial skepticism and the experimental nature of rTMS at that time. Limited access to advanced equipment, higher costs, and the need for robust clinical trials could have slowed its adoption [30]. However, the surge in publications in the last three years indicates an increasing number of publications, indirectly indicating higher research or scientific dissemination on rTMS in stroke [31].

The dominance of research articles as the primary publication type underscores the empirical foundation of this field, but the relatively smaller proportion of reviews and meta-analyses suggests a potential gap in synthesizing the rapidly growing body of evidence. This gap could hinder the integration of findings into clinical practice. Future efforts should emphasize producing high-quality systematic reviews, meta-analyses, and clinical guidelines that translate research findings into actionable insights for practitioners [32].

The global distribution of studies highlights China's leadership in rTMS research. This finding is not corroborative of the report that analyzed data up to 2023 by Li et al. [6]. This may be due to China's substantial government investment in neuroscience and technology. In contrast, other countries such as India and Brazil, despite their vast potential, contribute relatively little. This disparity might stem from resource limitations, insufficient research infrastructure, or lower prioritization of neurorehabilitation. International collaborations, capacity-building initiatives, and funding support could help underrepresented regions increase their contribution to this field. Additionally, fostering cross-border partnerships could bring diverse perspectives and accelerate innovation [33].

The keyword analysis reveals a cohesive research focus on stroke and neuromodulation, with emerging terms such as theta-burst stimulation reflecting the field's progression toward innovative approaches [34]. However, the limited occurrence of terms related to patient-centered outcomes, long-term efficacy, and healthcare accessibility points to areas requiring greater attention. Future research should emphasize these aspects to ensure that advancements in rTMS translate into meaningful improvements in patient care and health equity [35].

The influence of key studies and authors in shaping the field is evident from the citation data and author network analysis. However, the prominence of a few researchers such as Abo et al. suggests the potential risk of over-reliance on a small group of experts. Expanding mentorship opportunities and fostering collaboration with emerging researchers could diversify contributions and enhance the field's resilience [36]. Initiatives to increase the visibility of underrepresented authors and regions may also democratize the research landscape.

The concentration of publications in specific journals such as Frontiers in Neurology and Stroke underscores the targeted dissemination of findings to audiences most engaged with neurorehabilitation. However, others journal collectively contribute more than 50% of the publications. Although establishing specialized, high-impact journals dedicated to neuromodulation and stroke rehabilitation could provide a focused platform for researchers and practitioners alike, its wider dissemination may be achieved by publication in a diverse range of medical journals.

The dominance of major publishers such as Elsevier and Frontiers Media SA may suggest that these platforms provide the preferred avenues for disseminating impactful research. Their broad reach and influence might attract high-quality studies, but the decline in contributions from smaller publishers points to possible challenges in competing for impactful content. This unequal distribution highlights the need for strengthening publication opportunities in niche or emerging platforms to promote diversity in research dissemination [37]. Additionally, strategies such as open-access initiatives and collaborations with less prominent journals could ensure more equitable knowledge sharing.

The decentralized institutional landscape, with a significant portion of publications from "other" institutions, highlights the inclusivity of this research domain. However, the relatively small output from leading institutions, such as Harvard University raises questions about the broader dissemination of expertise and resources. Building international networks and encouraging collaborative projects across academic centers of excellence could strengthen the field. Moreover, promoting knowledge exchange between high-performing institutions and smaller or emerging research centers may further diversify contributions [38]. The study's novelty lies in its up-to-date bibliometric analysis of rTMS in stroke rehabilitation, offering unique insights into publication trends, influential authors, emerging themes, and regional contributions till 2024. For more robust studies related to rTMS in stroke, only the title was searched in WOS. This ensures the exclusion of false positive results. It identifies key research articles only. However, its limitations include reliance on one database and bibliometric indicators, which may not reflect all articles that may not be indexed in WOS but indexed in other databases.

## Conclusions

The bibliometric analysis of rTMS in stroke rehabilitation reveals a rapidly growing research landscape. Publications have substantially increased in recent years, with research articles predominantly representing the studies across clinical neurology, neuroscience, and rehabilitation domains. The People's Republic of China, South Korea, the USA, and Japan emerged as key contributors, with Elsevier and Frontiers Media leading publication efforts. These findings underscore the promising potential of rTMS in stroke recovery while emphasizing the need for more diverse geographical research and international collaboration.
